# Twenty-Three-Year Mortality in Parkinson’s Disease: A Population-Based Prospective Study (NEDICES)

**DOI:** 10.3390/jcm14020498

**Published:** 2025-01-14

**Authors:** Carla María Benito-Rodríguez, Félix Bermejo-Pareja, Ángel Berbel, José Lapeña-Motilva, Julián Benito-León

**Affiliations:** 1 Faculty of Medicine, Alfonso X el Sabio University, 28691 Madrid, Spain; cbenirod@myuax.com (C.M.B.-R.); angel.berbel@salud.madrid.org (Á.B.); 2 Instituto de Investigación Sanitaria Hospital 12 de Octubre (imas12), 28041 Madrid, Spain; fbermejop@h12o.es (F.B.-P.); lapena@salud.madrid.org (J.L.-M.); 3 Department of Neurology, Hospital Central de la Cruz Roja, 28003 Madrid, Spain; 4 Department of Neurology, University Hospital “12 de Octubre”, 28041 Madrid, Spain; 5 Department of Medicine, Faculty of Medicine, Complutense University, 28040 Madrid, Spain; 6 Centro de Investigación Biomédica en Red Sobre Enfermedades Neurodegenerativas (CIBERNED), 28031 Madrid, Spain

**Keywords:** Parkinson’s disease, mortality, population-based study, risk factors, aging

## Abstract

**Background:** Parkinson’s disease (PD) is one of the most prevalent neurodegenerative disorders among older adults, yet its long-term impact on mortality within population-based cohorts remains insufficiently characterized. This study leverages data from the Neurological Disorders in Central Spain (NEDICES) cohort to provide a comprehensive 23-year mortality analysis in a Spanish population. **Methods:** In this prospective cohort study, 5278 individuals aged 65 years and older were evaluated across two waves: baseline (1994–1995) and follow-up (1997–1998). At baseline, 81 prevalent PD cases were identified, while 30 incident cases, likely in the premotor phase at baseline, were detected during follow-up. Mortality was tracked over 23 years, and Cox proportional hazard models were employed to estimate hazard ratios (HRs) for mortality, adjusting for relevant demographic and clinical variables. **Results:** Fifty-three individuals from the cohort in the reference group (without PD) were excluded due to unreliable mortality data. Among 111 PD cases, 109 (98.2%) died during follow-up compared to 4440 (86.8%) of 5114 without the disease. PD was associated with a significantly increased mortality risk (adjusted HR = 1.62; 95% confidence interval [CI] = 1.31–2.01). Patients with both PD and dementia had an even higher risk (HR = 2.19; 95% CI = 1.24–3.89). Early-onset PD (<65 years) showed heightened mortality risk (HR = 2.11; 95% CI = 1.22–3.64). Cardiovascular and cerebrovascular diseases were the leading causes of death in both PD and non-PD participants. PD was significantly more often listed as the primary cause of death in PD patients compared to the reference group (14.7% vs. 0.4%, *p* < 0.001). **Conclusions:** PD significantly increases mortality risk over 23 years, particularly among those with early onset and dementia. These findings underscore the importance of a multidisciplinary approach to PD care, targeting both motor and non-motor symptoms to enhance long-term outcomes.

## 1. Introduction

Parkinson’s disease (PD) affects over 1% of individuals aged 60 years and older, with prevalence increasing to approximately 5% in those aged 85 and above [1]. This burden is especially significant in aging populations globally, fueled by demographic transitions and increasing life expectancy [1]. While PD is primarily characterized by motor symptoms such as bradykinesia, rigidity, and resting tremor, it also encompasses a wide range of non-motor symptoms, including cognitive impairment and autonomic dysfunction, which significantly contribute to both morbidity and mortality [1,2,3,4].

Autonomic dysfunction in PD is particularly significant, as it interacts with cognitive impairment and motor symptoms, adding further complexity to disease management [3]. However, dysautonomia is not exclusive to PD; it is also a prominent feature of other atypical parkinsonian syndromes, such as multiple system atrophy and progressive supranuclear palsy [3]. These overlapping features underscore the need for comprehensive diagnostic criteria to distinguish PD from related disorders accurately. To address this, the International Parkinson and Movement Disorder Society (MDS) introduced new clinical diagnostic criteria for PD in 2015, aiming to improve diagnostic precision by integrating both motor and non-motor features [5].

Meta-analyses consistently confirm an elevated mortality rate among PD patients compared to the general population [2], with advanced age, male sex, and the presence of dementia consistently identified as significant predictors of mortality across studies [2,6,7]. However, significant variability in reported rates reflects critical gaps in understanding of the disease. Differences in methodology, population heterogeneity, and follow-up durations complicate direct comparisons. Moreover, clinical cohorts often depend on hospital-based registries, which may underrepresent milder or undiagnosed cases, potentially underestimating the overall burden of PD [8,9].

In contrast, population-based studies offer a more comprehensive perspective by including a broader range of disease severities and capturing undiagnosed cases [2,8,9]. Such studies are crucial for accurately assessing the true mortality burden of PD and understanding its long-term impact. Table 1 summarizes key findings from previous population-based studies [7,10,11,12,13,14,15,16,17,18,19,20,21,22,23].

The current study leverages the Neurological Disorders in Central Spain (NEDICES) cohort, a large, prospective, population-based sample with neurologist-confirmed diagnoses, to address these gaps [24,25]. Previous analyses of the NEDICES cohort during a 13-year follow-up demonstrated a significantly elevated mortality risk in PD, with an adjusted hazard ratio (HR) of 1.75 compared to controls, and identified dementia as a significant risk factor [7]. However, given PD’s insidious progression and its impact on aging populations, further longitudinal analyses are needed to capture long-term determinants of mortality.

We hypothesize that PD remains an independent risk factor for mortality over an extended 23-year follow-up, with dementia and early-onset disease playing prominent roles in increasing mortality risk. By extending the follow-up of the NEDICES cohort, this study provides robust, population-based evidence on mortality trends and survival determinants in PD over two decades. These findings may contribute to a deeper understanding of PD progression, inform clinical strategies, and support public health efforts to address the needs of aging populations affected by this complex disease.

## 2. Methods

### 2.1. Study Areas

**Figure 1:**
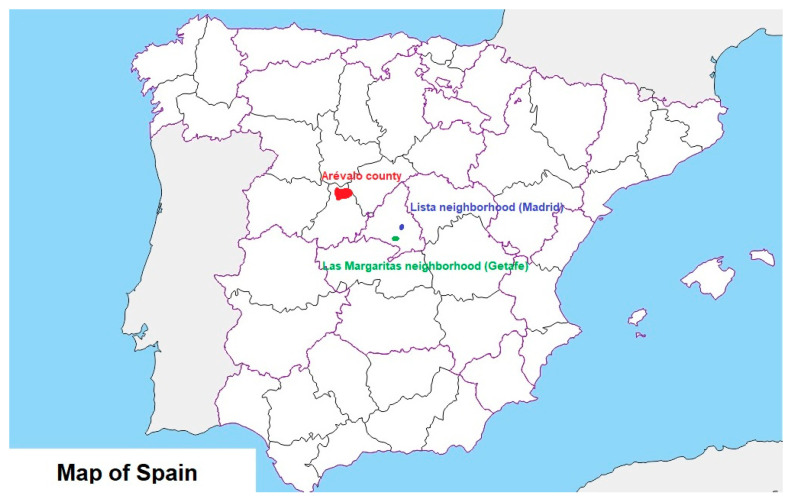
Geographic location of the three study areas in the NEDICES study: Arévalo county (red), a rural area in Ávila; Lista neighborhood (blue), a middle- to upper-class urban area in Madrid; and Las Margaritas neighborhood (green), a working-class urban area in Getafe.

The NEDICES study was conducted in three distinct regions in central Spain: two urban neighborhoods (Lista in Madrid, a middle- to upper-class area, and Las Margaritas in Getafe, a working-class area) and a rural region (Arévalo county in Ávila) (Figure 1). These areas were selected for their varied socioeconomic profiles, suitable population sizes for studying neurological disease prevalence, availability of computerized medical records, and the support of the University Hospital “12 de Octubre,” in Madrid, which provided consistent access to neurologists.

### 2.2. Study Design

The baseline assessment, conducted between 1994 and 1995, included 5278 individuals aged 65 and older. It targeted various neurological conditions to determine the prevalence of several age-related diseases, including dementia, essential tremor, PD and other parkinsonian syndromes, and cerebrovascular diseases [7,24,25,26,27,28,29].

The NEDICES study employed a two-phase door-to-door design to ensure comprehensive case capture within the target population [24,25].

Screening phase: During the baseline (1994–1995) and follow-up (1997–1998) assessments, participants were interviewed using a structured questionnaire to collect demographic information, medication use, medical history, smoking habits (ever vs. never), and alcohol consumption (ever/at least once per week vs. never) [24,25]. When participants could not be reached in person, a shortened version of the questionnaire was mailed. Family members provided information when participants were unavailable, while family physicians supplemented details as needed. A specific questionnaire was used to record information about deaths where applicable.

Specific parkinsonism screening questions were incorporated into both the baseline (1994–1995) and follow-up (1997–1998) assessments [28,30]. These questions included a history of PD diagnosis, the presence of tremor, and slow walking, adapted from the Italian Longitudinal Study on Aging [31]. To evaluate the effectiveness of the screening process, a randomly selected sample of 183 individuals who initially screened negative for parkinsonism underwent detailed examinations. None of these participants exhibited signs of parkinsonism, confirming the effectiveness of the screening questions [32].

Diagnostic phase: Participants who screened positive for neurological symptoms, including parkinsonism-related questions, were referred to Phase 2 for a detailed neurological evaluation. A single affirmative response to parkinsonism screening questions was sufficient to warrant further evaluation. Parkinsonism was diagnosed if at least two of the four cardinal signs (resting tremor, rigidity, bradykinesia, and postural reflex impairment) were present or if a single cardinal sign was observed in participants on antiparkinsonian medication.

Neurologists conducted thorough evaluations using the Unified Parkinson’s Disease Rating Scale (UPDRS) to assess bradykinesia, tremor, rigidity, gait abnormalities, and postural instability [33]. Cases were categorized as drug-induced parkinsonism, vascular parkinsonism, or PD. Parkinsonism linked to other conditions—such as nervous system infections, significant head trauma, brain tumors, or other neurological disorders potentially impacting the basal ganglia—was identified through routine clinical evaluation. This category also encompassed Parkinson-plus syndromes. Subjects were classified as having definite PD or idiopathic parkinsonism after ruling out all other potential causes of parkinsonism. To ensure diagnostic accuracy, a panel of four senior neurologists reviewed all cases and resolved any discrepancies through consensus.

Dementia status was determined using the criteria outlined in the Diagnostic and Statistical Manual of Mental Disorders, Fourth Edition (DSM-IV) [34]. PD with dementia was diagnosed in cases where cognitive decline occurred after the onset of motor symptoms and was associated with PD progression. The severity of PD was classified according to the Hoehn and Yahr scale [35].

Mortality was tracked until 31 December 2017. Death dates and causes were obtained from Spain’s National Population Register. Death certificates issued by attending physicians were categorized using ICD-10 codes, with cases initially coded under ICD-9 recoded to ICD-10 by neurologists and a statistician to ensure consistency. The principal underlying cause of death was determined as the condition initiating the sequence leading to death. Causes were classified into six main categories: PD, dementia, cardiovascular and cerebrovascular diseases, respiratory disease, cancer, and other causes (including infections, trauma, and genitourinary or gastrointestinal disorders).

### 2.3. Statistical Analyses

Data analyses were conducted using SPSS version 29.0 and Python 3.12.2, with pandas 2.2.2 and lifelines 0.29.0. All *p*-values were two-tailed, with significance set at *p* < 0.05. Categorical variables were analyzed using the chi-squared test. Continuous data were assessed for normality using the Kolmogorov–Smirnov test, which revealed that the data were not normally distributed. Consequently, nonparametric analyses were employed for statistical comparisons, ensuring the appropriate handling of the data distribution.

A comorbidity score was calculated based on conditions like atrial fibrillation, cancer, chronic obstructive pulmonary disease, depression, dementia, diabetes, heart failure, myocardial infarction, psychiatric disorders, renal disease, and stroke, producing a cumulative score from 0 (no conditions) to 28 (all conditions present) [36]. This cumulative score was used as a covariate in the statistical models to adjust for the impact of comorbidities on mortality risk.

Cox proportional hazard models were used to estimate HRs for mortality, accompanied by 95% confidence intervals (CI). The time to event was defined as years from the baseline assessment (1994–1995) to either 31 December 2017 for survivors or the date of death for deceased participants. Cox models were developed incrementally, starting with an unadjusted model, followed by models adjusting for variables significantly associated with both PD and mortality (strict criterion) or either PD or mortality (less strict criterion), and finally, a fully adjusted model. Kaplan–Meier survival curves compared PD cases to controls, with the log-rank test assessing differences between groups.

## 3. Results

### 3.1. Study Population

**Figure 2:**
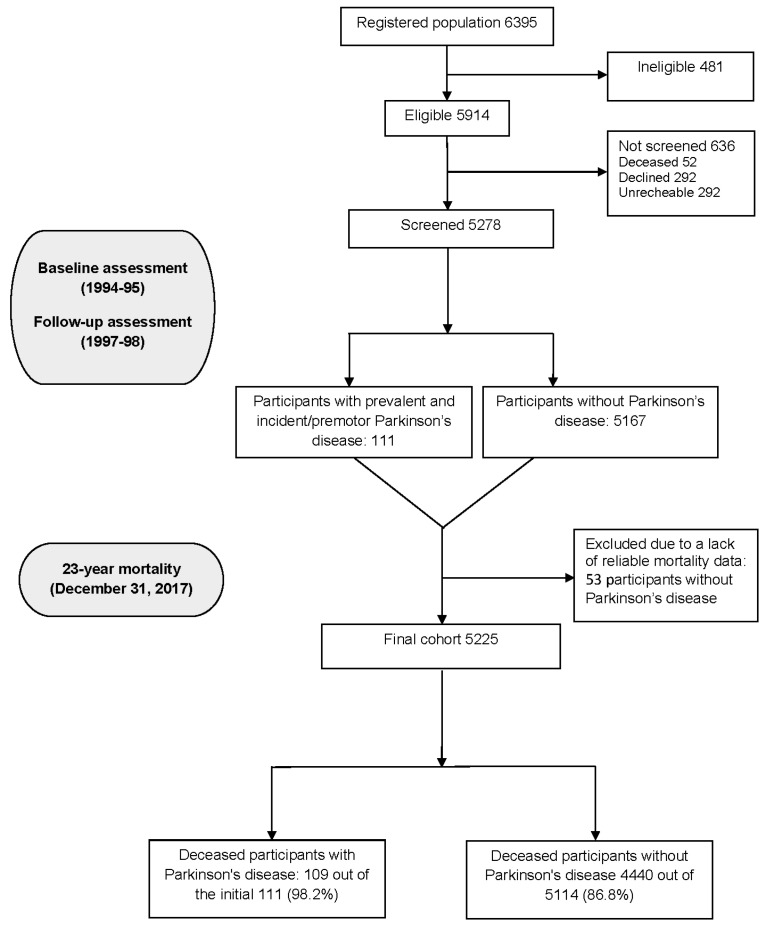
Flowchart of the study.

Starting in January 1994, letters explaining the survey and inviting participation were sent to 6395 individuals. Of these, 5914 were deemed eligible for screening, and 5278 of the 5914 eligible individuals (89.2%) were evaluated. Among the 636 individuals who were not evaluated, 292 (45.9%) declined participation, 292 (45.9%) could not be located due to a change in address, and 52 (8.2%) had passed away (Figure 2).

In this study, premotor PD patients—diagnosed during follow-up (1997–1998) but not at baseline (1994–1995) and likely representing early-stage cases—were included in the baseline PD group. This decision aligns with emerging evidence suggesting that non-motor symptoms may represent an early stage of the disease [37,38]. Research increasingly supports a progression from non-motor to motor symptoms, highlighting PD as a continuum [39,40]. Consequently, including these cases aligns with the evolving understanding of the disease’s progression and spectrum [39,40].

The baseline cohort comprised 5278 participants, including 81 with PD, 30 likely in the premotor phase at baseline—diagnosed with PD during follow-up—and 5,167 without PD (control group) (Figure 2) [28,30]. However, 53 individuals in the cohort, all of whom were in the control group, were excluded due to the lack of reliable mortality data. This exclusion was considered to have minimal impact on the overall findings, given its small proportion (1.0%) and the fact that it did not affect the PD group. Consequently, the study cohort consisted of 111 PD patients and 5114 in the reference group (total = 5225) (Figure 2).

### 3.2. Baseline Characteristics

Among the 81 prevalent PD patients, 3 (3.7%) had lived with the disease for 20 years or more, 19 (23.4%) for 10–19 years, and 59 (72.8%) for 1–9 years. The age of onset ranged from 41 to 84 years, with a median of 70 years. Hoehn & Yahr staging was distributed as follows: stage I (9 patients, 11.1%), stage II (40 patients, 49.4%), stage III (12 patients, 14.8%), stage IV (16 patients, 19.8%), and stage V (4 patients, 4.9%). Of these 81 patients, 61 (75.3%) were examined by NEDICES neurologists, while the remaining 20 cases were diagnosed based on medical report reviews. Notably, 23 PD patients (28.4%) had not been diagnosed prior to the survey. Among the 58 previously diagnosed patients, all had shown a positive response to levodopa.

The median follow-up period for the cohort was 12.2 years (range: 0.03–23.9 years); during this time, 4549 (87.1%) of the 5225 participants died. Among the 111 PD cases (prevalent and incident), 109 (98.2%) died during follow-up, while 4440 (86.8%) of the 5114 without PD disease died during the same period (Figure 2).

There were significant differences in age and medical comorbidities between PD patients and those without PD. Additionally, the proportion of men and individuals with dementia was higher in the PD group. (Table 2). Furthermore, participants who died were older, more likely to be male, had lower education levels, higher rates of smoking, a greater prevalence of arterial hypertension, a higher comorbidity burden, and were more frequently from the Arévalo area compared to those who survived (Table 3).

### 3.3. Follow-Up and Mortality Outcomes

In an unadjusted Cox model, PD patients had a significantly higher mortality risk (HR = 1.91, 95% CI = 1.58–2.32, *p* < 0.001) compared to those without PD (reference group). After adjusting for age, sex, and the comorbidity index—factors associated with both PD and mortality—the elevated risk remained significant (HR = 1.56, 95% CI = 1.29–1.89, *p* < 0.001, Model 1 in Table 4A). This increased risk persisted in a Cox model adjusted for factors associated with either PD or mortality (age, sex, study area, education, smoking status, arterial hypertension, and comorbidity index; Model 2, *p* < 0.001) and in a model adjusting for all variables (Model 3, *p* < 0.001) (Table 4A).

In additional Cox models, we observed an increased mortality risk among individuals with both PD and dementia, as well as those with PD without dementia, compared to the reference group (see Table 4B). Specifically, the HR for PD patients with dementia was more than double compared to those with PD without dementia. The adjusted HRs were consistently higher for PD patients with dementia across all models, highlighting dementia as a significant factor contributing to mortality risk in PD.

Table 4C presents mortality HRs in PD patients stratified by age at disease onset, showing that those diagnosed with earlier-onset PD (before age 65) had a higher mortality risk compared to individuals with later-onset PD.

### 3.4. Survival Curves

**Figure 3:**
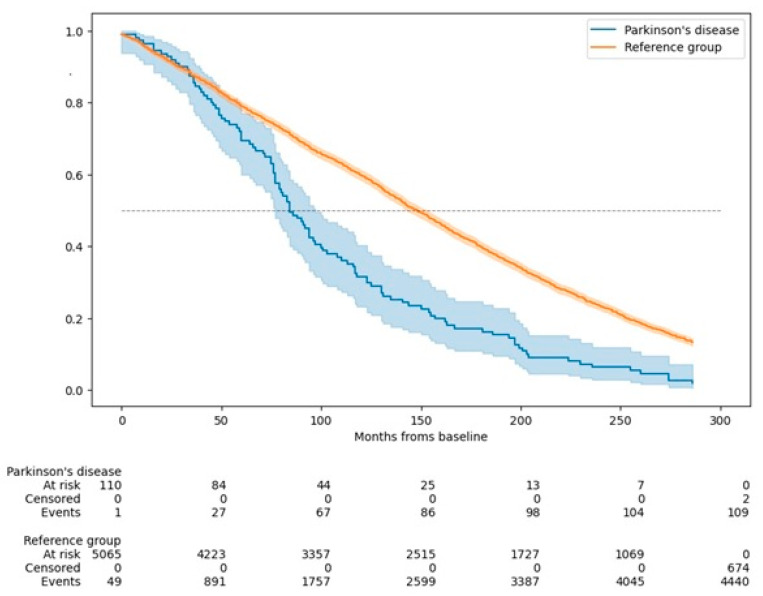
Kaplan–Meier curves showing the overall survival of the NEDICES cohort.

The analysis depicted in Figure 3 shows a marked difference in survival between PD patients and those in the reference group. The survival curve for the PD group declines more rapidly, indicating a significantly reduced survival probability among PD patients (log-rank test [Mantel–Cox]: χ^2^ = 46.247, *p* < 0.001). By the study’s conclusion, nearly all participants with PD had passed away, whereas a considerable portion of the reference group remained alive.

The estimated mean survival time for individuals without PD was 12.7 years (95% CI: 12.5–12.9 years), with a median survival time of 12.4 years (95% CI: 12.0–12.7 years). In contrast, PD patients had a substantially lower estimated mean survival time of 8.8 years (95% CI: 7.7–9.9 years) and a median survival time of 7.1 years (95% CI: 6.1–8.0 years).

### 3.5. Causes of Death

Notably, PD itself was a significantly more common cause of death in the PD group, with 14.7% of deaths attributed to it, compared to only 0.4% in those without PD (*p* < 0.001) (Table 5). No significant differences were found for other causes of death, including dementia, cardiovascular and cerebrovascular diseases, respiratory diseases, cancer, and other causes (Table 5).

## 4. Discussion

To our knowledge, this is among the first prospective, population-based studies with over two decades of follow-up where neurologists directly evaluated PD patients. This rigorous methodology ensures high diagnostic precision and provides robust insights into long-term mortality outcomes, complementing earlier studies such as the Honolulu Heart Study [12] while offering a broader perspective through a diverse population-based sample. Our findings reveal a significantly increased 23-year mortality risk among older adults with PD in Spain, with these individuals experiencing substantially higher mortality rates than those without the disease, even after adjusting for multiple confounders.

### 4.1. Key Findings

In the initial model, adjusting for age, sex, and comorbidity index yielded an HR of 1.56 (95% CI = 1.29–1.89), which remained significantly elevated at 62% (HR = 1.62, 95% CI = 1.31–2.01) in the fully adjusted model. These findings align with prior studies linking PD to elevated mortality [2], though our extended 23-year follow-up provides a uniquely comprehensive perspective on long-term mortality trajectories. This extended duration offers valuable insights into disease progression and mortality beyond the typical follow-up periods of most studies. The prospective, population-based design of the NEDICES study, combined with neurologist-confirmed diagnoses, minimizes selection bias and enhances both the accuracy and generalizability of our findings, establishing this study as a significant contribution to the understanding of PD mortality.

The consistency of our results with those from the earlier 13-year NEDICES mortality analysis reinforces the validity of our findings [7]. At 13 years, the adjusted HR for mortality in PD patients was 1.75 (95% CI = 1.32–2.31) [7], closely aligning with our current 23-year outcome. This stability over time suggests that the elevated mortality risk associated with PD remains steady, even with prolonged follow-up.

Xu et al.’s [2] meta-analysis reported a higher all-cause mortality risk for PD patients (relative risk = 2.22; 95% CI = 1.78–2.77). In contrast, our adjusted 23-year HR is slightly lower than those reported in other population-based studies (Table 1). This difference may be explained by our door-to-door, population-based methodology, which likely captured a broader spectrum of PD cases, including milder forms often underrepresented in studies relying solely on clinical records. These milder cases, with less severe disease progression, may have contributed to the comparatively lower HR in our cohort.

Additionally, the inclusion of premotor (incident) PD cases in our analysis may have contributed to the slightly lower mortality risk observed compared to studies focusing exclusively on patients with manifest motor symptoms (Table 1). Premotor cases represent earlier and less severe stages of PD, where mortality risks are less pronounced [39,40]. Emerging research underscores the value of advanced imaging techniques, such as positron emission tomography, in identifying premotor PD markers, including rapid eye movement sleep behavior disorder [41]. Although our study did not incorporate imaging data to detect premotor cases, incident PD patients identified during the follow-up (1997–1998) were diagnosed using the same rigorous methodology as prevalent cases, ensuring minimal case loss. By including premotor (incident) cases, we aimed to provide a more comprehensive understanding of disease progression and its long-term mortality implications.

On the other hand, our extended follow-up may have diluted the observed mortality impact, as studies with follow-up periods longer than ten years often show lower relative risks [2]. Advances in PD treatment and management over the study period may also have contributed to reducing excess mortality, though further research is needed to confirm this hypothesis. Finally, population-specific factors may influence PD survival; unique characteristics of the Spanish population could partly explain the lower HR observed in our study compared to studies from other regions.

### 4.2. Impact of Dementia

Dementia within the context of PD emerged as a particularly significant factor, with individuals experiencing both conditions showing the highest mortality risk (adjusted HR = 2.19, 95% CI = 1.24–3.89). This finding is consistent with previous studies that have identified dementia as a critical predictor of mortality in PD [18,42], including the earlier 13-year NEDICES analysis [7], which also reported a notably high HR in this subgroup. The association between dementia and increased mortality risk in PD may be attributed to several mechanisms, including an elevated risk of falls and associated complications [43], poorer treatment adherence [44], and possibly an indication of a more aggressive or advanced form of PD [45,46]. Our findings underscore the importance of close monitoring and comprehensive management of cognitive impairment in PD patients, particularly given the strong association between dementia and mortality risk.

### 4.3. Age of Onset and Mortality

Our study identified a compelling trend observed in other research [23], namely that individuals with early-onset PD (diagnosed before age 65) exhibited a heightened mortality risk (adjusted HR = 2.11, 95% CI = 1.22–3.64) compared to those with later-onset PD. Early-onset PD may lead to prolonged exposure to PD-related complications, potentially increasing overall mortality risk. This finding contrasts with other studies that have associated early-onset PD with more favorable long-term outcomes [4,47,48]. However, much of the prior research on early-onset PD is based on data from patients in specialist clinic settings, which may limit the generalizability of these results to the broader population. Further research is, however, needed to explore the distinct characteristics and risk factors associated with early-onset PD.

### 4.4. Underlying Causes of Mortality

Regarding causes of death, it is noteworthy that PD was identified as the primary cause in only 14.7% of cases. Cardiovascular and cerebrovascular diseases emerged as the most frequently reported causes of death in both PD and non-PD individuals, aligning with findings from the 13-year NEDICES analysis and other hospital-based studies [7,49]. The low proportion of deaths directly attributed to PD likely reflects the underreporting of the condition on death certificates—a well-recognized limitation in PD mortality studies that can lead to an underestimation of its true impact [21,50].

Distinguishing between direct complications of PD and comorbidities is particularly challenging in older populations, as the disease increases susceptibility to other conditions [51,52], indirectly contributing to mortality. Although our study found no significant differences in cardiovascular and cerebrovascular mortality between PD patients and controls, Hong et al.’s [53] meta-analysis identified an elevated risk of these conditions in PD, potentially linked to shared pathogenetic mechanisms. These findings highlight the importance of prioritizing vascular health in PD management to improve long-term outcomes.

### 4.5. Strengths and Limitations

Our study has several important strengths. The population-based design reduces selection bias often observed in hospital-based studies, while the extended 23-year follow-up provides a unique long-term perspective on PD mortality trajectories. The inclusion of a broad range of covariates allows for robust adjustments for potential confounders, and the use of a validated comorbidity index strengthens the reliability of our findings compared to studies employing fewer specific indices. Additionally, the continuity with the earlier 13-year NEDICES analysis facilitates direct comparisons and enables the evaluation of trends over time.

However, certain limitations should be acknowledged. First, relying on initial assessments for diagnoses meant that we could not capture all incident PD cases during the 23-year follow-up, which may have led to an underestimation of PD-related mortality risk. Second, we lacked detailed information on treatment methods over the study period, making it difficult to assess how therapeutic advancements may have influenced mortality outcomes. Third, the absence of systematic assessment for disease subtypes precluded an analysis of how specific PD subtypes (e.g., tremor-dominant vs. akinetic rigid dominant subtype subtypes) might influence survival trajectories.

Finally, the potential for misdiagnosis, particularly with clinically similar conditions such as progressive supranuclear palsy-parkinsonism predominant, should be considered [54]. Despite neurologist-confirmed diagnoses aimed at minimizing misclassification, the lack of advanced imaging and specific diagnostic tools at the time of data collection may have led to diagnostic overlap. Progressive supranuclear palsy-parkinsonism predominant, for instance, shares several motor and non-motor features with PD but differs in progression and prognosis [54]. This limitation underscores the need for integrating more sophisticated diagnostic criteria and tools, such as advanced imaging techniques and biomarkers, to differentiate PD from atypical parkinsonian syndromes more effectively in future studies [55,56,57].

### 4.6. Conclusions

The findings from our study have important implications for clinical practice, research, and public health. Effective PD management requires addressing motor symptoms, cognitive impairment, and comorbidities. Identifying mortality risk factors, such as cognitive decline, can help clinicians stratify patients by risk, enabling personalized treatment plans and improved outcomes.

Our results emphasize the need for developing precise prognostic models to guide clinical decisions and resource allocation. Future research should explore the mechanisms underlying increased mortality in early-onset PD, evaluate the long-term effects of treatment strategies, and investigate the relationship between PD and vascular disease. Incorporating biomarkers and genetic data could further clarify variability in disease progression and its impact on survival [55,56,57].

As PD prevalence rises with an aging population, effective healthcare planning will be critical. A multidisciplinary approach that includes managing cognitive and vascular complications is essential to improve survival and quality of life for this expanding patient population. These efforts will support better patient care and inform policies to address the growing burden of PD.

## Figures and Tables

**Table 1:** Summary of mortality risk in Parkinson’s disease: key findings from a selection of major population-based studies.

Year	Study	Cohort Characteristics	Key Findings
1990	Ebmeier et al. [10]	267 patients and 233 matched controls in Scotland were followed for 3.5 years	The relative mortality rate was 2.35. Factors predicting death included cognitive impairment, older age, late disease onset, long-term smoking, low blood pressure, and Parkinson’s-related mobility issues
1995	Ben-Shlomo and Marmot [11]	220 parkinsonian patients and 421 matched controls in the UK were followed for 20 years	The all-cause hazard ratio for patients compared with controls was 2.6. Both ischemic heart disease (2.3) and cerebrovascular disease (3.6) showed significantly increased hazard ratios
1996	Morens et al. [12]	8006 middle-aged men from the Honolulu Heart Study were followed for 29 years	Between the ages of 70 and 89, Parkinson’s disease patients had a two- to three-fold increase in the mortality risk (mortality ratio = 2.5); survival was reduced by 8 years compared to controls
1997	Louis et al. [13]	288 Parkinson’s disease patients, Manhattan, USA	The mortality risk, when compared with nondemented elderly subjects, was highest among those with both Parkinsón’s disease and dementia (rate ratio = 4.9). Dementia in the absence of Parkinson’s disease also was associated with an increased mortality risk (rate ratio = 1.6). A high baseline total extrapyramidal signs score was associated with significantly earlier mortality
2000	Berger et al. [14]	Pooled analysis of five European population-based cohorts (16,143 participants, including 252 with Parkinson’s disease)	The relative risk of death in Parkinson’s disease = 2.3. The risk for death in men with Parkinson’s disease (relative risk = 3.1) was higher than in women (relative risk = 1.8)
2000	Donnan et al. [15]	97 Parkinson’s disease patients in Scotland	Parkinson’s disease patients, in relation to comparators, had higher mortality with a rate ratio of 1.76 in the 7-year cohort. There was significantly greater mortality in PD patients who received levodopa monotherapy (rate ratio = 2.45)
2000	Morgante et al. [16]	59 patients and 118 matched controls in Sicily, Italy, were followed for 8 years.	Parkinson’s disease mortality was significantly higher, with a relative risk of 2.3; pneumonia was the most common cause of death
2003	Fall et al. [17]	170 Parkinson’s disease patients and 510 matched controls in Sweden were followed for 9.4 years	The mortality rate ratio was 1.6 when comparing Parkinson’s disease patients with controls, and the all-cause hazard ratio was 2.4. PD patients also experienced a significantly higher proportion of deaths due to pneumonia
2005	de Lau et al. [18] (Rotterdam Study)	6969 participants, including 99 prevalent and 67 incident Parkinson’s disease patients	Increased mortality risk (hazard ratio = 1.83). Within PD patients, mortality risk was influenced by disease duration and by the occurrence of dementia
2010	Forsaa et al. [19]	230 Parkinson’s disease patients, followed from 1993 to 2009, Norway	Median survival was 15.8 years; mortality predictors included higher age at onset, older age, male sex, more severe motor impairment, psychotic symptoms, and dementia
2011	Posada et al. [7] (NEDICES Study)	5262 participants, including 81 Parkinson’s disease patients, followed over 13 years in Spain	Parkinson’s disease mortality was higher (adjusted hazard ratio = 1.75); dementia further increased the risk (adjusted hazard ratio = 2.60)
2017	Savica et al. [20]	461 patients with synucleinopathies, Minnesota, USA	Parkinson’s disease with dementia had a hazard ratio for death of 3.86. Parkinson’s disease alone had a hazard ratio of 1.75, while multiple system atrophy with parkinsonism exhibited the highest mortality, with a hazard ratio of 10.51
2018	Hobson and Meara [21]	166 Parkinson’s disease patients and 102 controls, followed for 18 years in Wales	Compared with the general UK population, Parkinson’s disease patients had a higher mortality risk, with a standardized mortality ratio of 1.82. The most common causes of death were pneumonia and cardiac-related conditions
2018	Keener et al. [22]	360 Parkinson’s disease patients in California with a mean follow-up period of 5.8 years	Lower baseline Mini-mental State scores, reported longer average sleep duration, greater motor symptom severity, and motor subtype (postural instability gait difficulty) were significant predictors of mortality
2019	Hoogland et al. [23]	133 newly diagnosed Parkinson’s disease patients in the Netherlands followed for at least 13 years	Increased mortality was associated with mild cognitive impairment, higher levodopa dose, and earlier onset

**Table 2:** Baseline demographic and clinical characteristics (1994–1995) of individuals with and without Parkinson’s disease—reference group—(N = 5225).

	Parkinson’s Disease (N = 111)	Without Parkinson’s (N = 5114)	*p* Value
Age in years	76.5 ± 5.7 (76.0)	74.3 ± 7.0 (73.0)	<0.001
Sex (female)	49 (44.1%)	2949 (57.7%)	0.004
Study area			0.359
- Arévalo	47 (42.3%)	1874 (36.6%)	
- Las Margaritas	31 (27.9%)	1725 (33.7%)	
- Lista	33 (29.6%)	1515 (29.6%)	
Education in years of study completed *	5.7 ± 7.1 (6.0)	6.0 ± 5.3 (6.0)	0.270
Smoking habit *			0.118
- Smoker	5 (5.4%)	489 (12.2%)	
- Ex-smoker	29 (31.5%)	1068 (26.7%)	
- Never smoked	58 (63.0%)	2436 (61.0%)	
Alcohol consumption *			0.233
- Regular drinker	23 (25.0%)	1334 (33.4%)	
- Ex-drinker	21 (22.8%)	830 (20.8%)	
- Never drank	48 (52.2%)	1825 (45.8%)	
Arterial hypertension *	62 (56.4%)	2483 (51.0%)	0.267
Comorbidity index	1.6 ± 1.8 (1.0)	1.1 ± 1.5 (0.0)	0.005

Mean ± standard deviation (median) values are provided for age, years of education, and comorbidity index. Mann–Whitney test (continuous data comparison) and chi-squared test (proportions) were used. * N < 5225 due to missing data for some participants.

**Table 3:** Baseline demographic and clinical characteristics (1994–1995) of the cohort, stratified by mortality (N = 5225).

	Alive (N = 676)	Deceased (N = 4549)	*p* Value
Age in years	68.9 ± 3.9 (68.0)	75.1 ± 7.0 (74.0)	<0.001
Sex (female)	460 (68.0%)	2538 (55.8%)	<0.001
Study area			0.006
- Arévalo	211 (31.2%)	1710 (37.6%)	
- Las Margaritas	249 (36.8%)	1507 (33.1%)	
- Lista	216 (32.0%)	1332 (29.3%)	
Education in years of study completed *	6.8 ± 5.7 (7.0)	5.9 ± 5.3 (6.0)	<0.001
Smoking habit *			0.002
- Smoker	62 (10.8%)	432 (12.3%)	
- Ex-smoker	125 (21.7%)	972 (27.7%)	
- Never smoked	388 (67.5%)	2106 (60.0%)	
Alcohol consumption *			0.050
- Regular drinker	208 (36.3%)	1149 (32.8%)	
- Ex-drinker	99 (17.3%)	752 (21.4%)	
- Never drank	266 (46.4%)	1607 (45.8%)	
Arterial hypertension *	254 (38.4%)	2291 (53.1%)	<0.001
Comorbidity index	0.6 ± 1.0 (0.0)	1.2 ± 1.6 (1.0)	<0.001

Mean ± standard deviation (median) values are provided for age, years of education, and comorbidity index. Mann–Whitney test (continuous data comparison) and chi-squared test (proportions) were used. * N < 5225 due to missing data in some participants.

**Table 4:** Mortality hazard ratios in Parkinson’s disease patients compared to non-PD participants and stratifications by dementia status and disease onset.

**A.** **Mortality Hazard Ratios in Parkinson’s Disease Patients Versus Those Without Parkinson’s Disease**												
	Unadjusted			Model 1			Model 2			Model 3		
	Hazard ratio	95% CI	*p* Value	Hazard ratio	95% CI	*p* Value	Hazard ratio	95% CI	*p* Value	Hazard ratio	95% CI	*p* Value
Parkinson’s disease patients (N = 111)	1.91	1.58–2.32	<0.001	1.56	1.29–1.89	<0.001	1.64	1.33–2.04	<0.001	1.62	1.31–2.01	<0.001
Participants without Parkinson’s disease (N = 5114) (reference group)	1.0	_	1.0	1.0	_	1.0	1.0	_	1.0	1.0	_	1.0
**B.** **Mortality hazard ratios in Parkinson’s disease patients, stratified by the presence or absence of dementia**												
	Unadjusted			Model 1			Model 2			Model 3		
	Hazard ratio	95% CI	*p* value	Hazard ratio	95% CI	*p* value	Hazard ratio	95% CI	*p* value	Hazard ratio	95% CI	*p* value
Parkinson’s disease patients with dementia (N = 14)	4.27	2.52–7.23	<0.001	2.13	1.26–3.62	0.005	2.19	1.24–3.88	0.007	2.19	1.24–3.89	0.007
Parkinson’s disease patients without dementia (N = 97)	1.77	1.44–2.17	<0.001	1.50	1.23–1.84	<0.001	1.58	1.26–1.99	<0.001	1.56	1.24–1.96	<0.001
Participants without either condition (N = 5114) (reference group)	1.0	_	1.0	1.0	_	1.0	1.0	_	1.0	1.0	_	1.0
**C.** **Mortality hazard ratios in Parkinson’s disease patients, stratified by disease onset**												
	Unadjusted			Model 1			Model 2			Model 3		
	Hazard ratio	95% CI	*p* value	Hazard ratio	95% CI	*p* value	Hazard ratio	95% CI	*p* value	Hazard ratio	95% CI	*p* value
Parkinson’s disease onset at age 65 or later (N = 92)	1.87	1.52–2.30	<0.001	1.46	1.19–1.80	<0.001	1.58	1.25–1.99	<0.001	1.56	1.24–1.97	<0.001
Parkinson’s disease onset before age 65 (N = 19)	2.16	1.38–3.40	<0.001	2.32	1.48–3.66	<0.001	2.15	1.25–3.71	0.006	2.11	1.22–3.64	0.007
Participants without Parkinson’s disease (N = 5114) (reference group)	1.0	_	1.0	1.0	_	1.0	1.0	_	1.0	1.0	_	1.0

Model 1 (baseline variables significantly associated with both Parkinson’s disease and mortality): adjusted for age, sex, and comorbidity index (atrial fibrillation, non-metastatic cancer, metastatic cancer, chronic obstructive pulmonary disease, depression, dementia, diabetes, treated epilepsy, heart failure, myocardial infarction, psychiatric disorders, kidney disease, and stroke). Model 2 (baseline variables significantly associated with either Parkinson’s disease or mortality): adjusted for age, sex, study area, education, smoking status, arterial hypertension, and comorbidity index. Model 3 (all variables): adjusted for age, sex, study area, education, smoking and alcohol status, arterial hypertension, and comorbidity index.

**Table 5:** Main cause of death (ICD-10) in individuals with and without Parkinson’s disease in NEDICES *.

Main Cause of Death	Parkinson’s Disease	Without Parkinson’s Disease	*p* Value **
Dementia	316 (7.2%)	7 (6.4%)	0.760
Cardiovascular and cerebrovascular diseases	1576 (35.8%)	30 (27.5%)	0.073
Parkinson’s disease	17 (0.4%)	16 (14.7%)	<0.001
Cancer	950 (21.6%)	17 (15.6%)	0.131
Respiratory diseases	660 (15.0%)	15 (13.8%)	0.719
Dementia	316 (7.2%)	7 (6.4%)	0.760
Other causes	879 (20.0%)	24 (22.0%)	0.601
Total	4398 (100%)	109 (100%)	

* The death cause of 42 participants could not be ascertained. ** Chi-square test.

## Data Availability

The original contributions presented in this study are included in the article. Further inquiries can be directed to the corresponding author.
